# Predicting soil erosion potential under CMIP6 climate change scenarios in the Chini Lake Basin, Malaysia

**DOI:** 10.1186/s40562-022-00254-7

**Published:** 2023-01-04

**Authors:** Muhammad Rendana, Wan Mohd Razi Idris, Sahibin Abdul Rahim, Zulfahmi Ali Rahman, Tukimat Lihan

**Affiliations:** 1 Department of Chemical Engineering, Faculty of Engineering, Universitas Sriwijaya, Indralaya, 30662 South Sumatra, Indonesia; 2 Department of Earth Science and Environment, Faculty of Science and Technology, Universiti Kebangsaan Malaysia, 43600 Bangi, Selangor Malaysia; 3 Department of Environmental Science, Faculty of Science and Natural Resources, Universiti Malaysia Sabah, 88400 Kota Kinabalu, Sabah Malaysia

**Keywords:** Climate change, Remote sensing, Revised universal soil loss equation, Soil loss

## Abstract

Climate change and soil erosion are very associated with environmental defiance which affects the life sustainability of humans. However, the potency effects of both events in tropical regions are arduous to be estimated due to atmospheric conditions and unsustainable land use management. Therefore, several models can be used to predict the impacts of distinct climate scenarios on human and environmental relationships. In this study, we aimed to predict current and future soil erosion potential in the Chini Lake Basin, Malaysia under different Climate Model Intercomparison Project-6 (CMIP6) scenarios (e.g., SSP2.6, SSP4.5, and SSP8.5). Our results found the predicted mean soil erosion values for the baseline scenario (2019–2021) was around 50.42 t/ha year. The mining areas recorded the highest soil erosion values located in the southeastern part. The high future soil erosion values (36.15 t/ha year) were obtained for SSP4.5 during 2060–2080. Whilst, the lowest values (33.30 t/ha year) were obtained for SSP2.6 during 2040–2060. According to CMIP6, the future soil erosion potential in the study area would reduce by approximately 33.9% compared to the baseline year (2019–2021). The rainfall erosivity factor majorly affected soil erosion potential in the study area. The output of the study will contribute to achieving the United Nations' 2030 Agenda for Sustainable Development.

## Introduction

Globalization has a prominent role in developing a country, such as Malaysia, an agricultural country due to its fertile soils and supported climate. In Malaysia, societies meet their daily needs with what they cultivate on fertile soils. The soil is a part of the ecosystem that can deal with a big hazard due to climate change events (Corwin 2021; Fahad et al. 2021; Rendana et al. 2019). For many years, the atmosphere of the earth has experienced a severe warming situation due to the increase of greenhouse gases (Shakoor et al. 2020; Manabe 2019). This leads to a high amount of rainfall with higher intensities and magnitude (Ohba and Sugimoto 2019). Panagos and Katsoyiannis (2019) explained the significance of the sustainable development goals (SDGs) on soil erosion. The SDGs goals are closely associated with land deterioration and assist to improve the relationship between soil, climate, and ecosystem functions. The integration of soil science and the SDGs can mitigate climate change, water scarcity, food scarcity, and biodiversity loss issues. Furthermore, the SDGs are generally presented on the earth system using three main pillars, such as environmental, economic, and social pillars (Purvis et al. 2019). All these aspects are closely associated with each other and implemented to gain long-term sustainability (Dalampira and Nastis 2020). The SDGs program aims to incorporate solutions according to ecological, social, and economical systems. Based on this program, zero land degradation will be attained by 2030 (Tóth et al. 2018).

Malaysia is a region with a good climate and riverine thus most fertile soils are formed due to precipitation, weathering, and pedo-geomorphological processes. However, recently, this important resource is in a very bad state. Soil erosion has become a nightmare for the people, especially for land developers. The severe impact of quick erosion can occur from natural and anthropogenic sources (Poesen 2018). Because of climate change and El-Niño impact on monsoon, the amount of rainfall increases with high intensity and short duration which leads to a higher risk of soil erosion (Xu et al. 2019; Zhu et al. 2019). Besides, misconduct in agricultural practices, tree logging, and building construction speed up the erosion process (Vijith et al. 2018). Therefore, a suitable condition not only induces soil erosion but also affects to cause flash floods (Diodato et al. 2022). To mitigate those issues, soil scientists have used a geographical component for the model to obtain the significant causatives for soil erosion (Rodrigo-Comino et al. 2018). Coupled SSPs–RCPs scenarios data showed a positive association between climate change and soil erosion (Li et al. 2021). Because the high amount of rainfall in a short duration can expedite the chemical weathering and produce more sediments.

The Universal Soil Loss Equation (USLE) method is extensively employed for calculating soil loss or erosion, since it can incorporate some factors of soil erosion (Alewell et al. 2019). Besides, the human factors that can promote soil erosion, current studies obtain other factors affecting land deterioration are climate (Xiong et al. 2019). Furthermore, the 4th scenario for the world’s greenhouse gases issue that is recognized as “Shared Socioeconomic Pathways” (SSPs) SSP2.6, SSP4.5, SSP7.0, SSP8.5 has been announced by IPCC (Intergovernmental Panel on Climate Change; Chen et al. 2020). These scenarios predict distinct greenhouse gas emission types, for instance, the SSP2.6 indicates low greenhouse gases emission, the SSP4.5, and SSP6 represent stable scenarios, whilst SSP8.5 expresses high greenhouse gases emission (Hu et al. 2021). Several works have been conducted to estimate the effects of clime on the erosion of soil using distinct CMIP5–RCP periods all over the world for instance, in Iran (Hateffard et al. 2021), Nepal (Talchabhadel et al. 2019), Turkey (Orozbaev et al. 2020), Sri Lanka (Senanayake and Pradhan 2022), Europe (Panagos et al. 2021), Uzbekistan (Gafforov et al. 2020), and Kenya (Watene et al. 2021). However, new studies about the effect of climate on the erosion of soil under CMIP6–SSP scenarios are still scarce. Therefore, in this current study, we would take focus on this topic.

Soil erosion is a native geological process; however, after anthropogenic interference may be categorized as a land quality reduction, which has been a repeated issue for long period across the world for authorities, and particularly, in nations, such as Malaysia (Rendana et al. 2018). Currently, researchers have investigated land deterioration factors in Malaysia, such as deforestation (Jaafar et al. 2020), soil acidification (Mahmud and Chong 2022), soil salinization (Kh’ng et al. 2021), land-use changes, and biodiversity loss (Wilkinson et al. 2018), and water erosion (Islam et al. 2020). Several studies have tried to combine geographic information systems, remote sensing, and the RUSLE method for the mapping of soil erosion (Anees et al. 2018; Roslee and Sharir 2019). Some current methods such as machine learning or artificial neural networks are also widely used for soil erosion prediction and mapping in Malaysia (Sarkar and Mishra 2018; Vu et al. 2020). However, in spite of the many studies on soil erosion analysis, there are narrow notions on soil erosion analysis according to future climate change scenarios in Malaysia and other tropical basin areas.

Chini lake basin, Malaysia has experienced significant economic growth over the 10-year period. Many land use activities in the basin area have converted from forests to oil palm rubber, mining, settlements, and tourism areas. These activities have greatly influenced the biological, hydrological, and ecological functions of the Chini lake ecosystem. Logging activities in the steep areas have led to severe environmental deterioration. Soil erosion rate, sedimentation, and nutrient loss have raised due to these changes. Pesticides and chemical fertilizers from agricultural activities have also raised N, P, and heavy metal concentrations in the lake water (Rendana et al. 2016). The soil erosion in the Chini basin area was sheet and rill erosions because of runoff events started by heavy rainfall. The bank erosion was found near the lake due to the effect of ripples produced by motorboats activities. These unsustainable land use systems around the basin area have led to various environmental issues especially soil erosion in the area. In addition, soil erosion would decline the terrestrial and aquatic biodiversities of the lake. Thus, the objective of this study was to predict the soil erosion potential in the Chini lake basin, Malaysia under CMIP6 scenarios.

## Materials and methods

### Study area

**Fig. 1:**
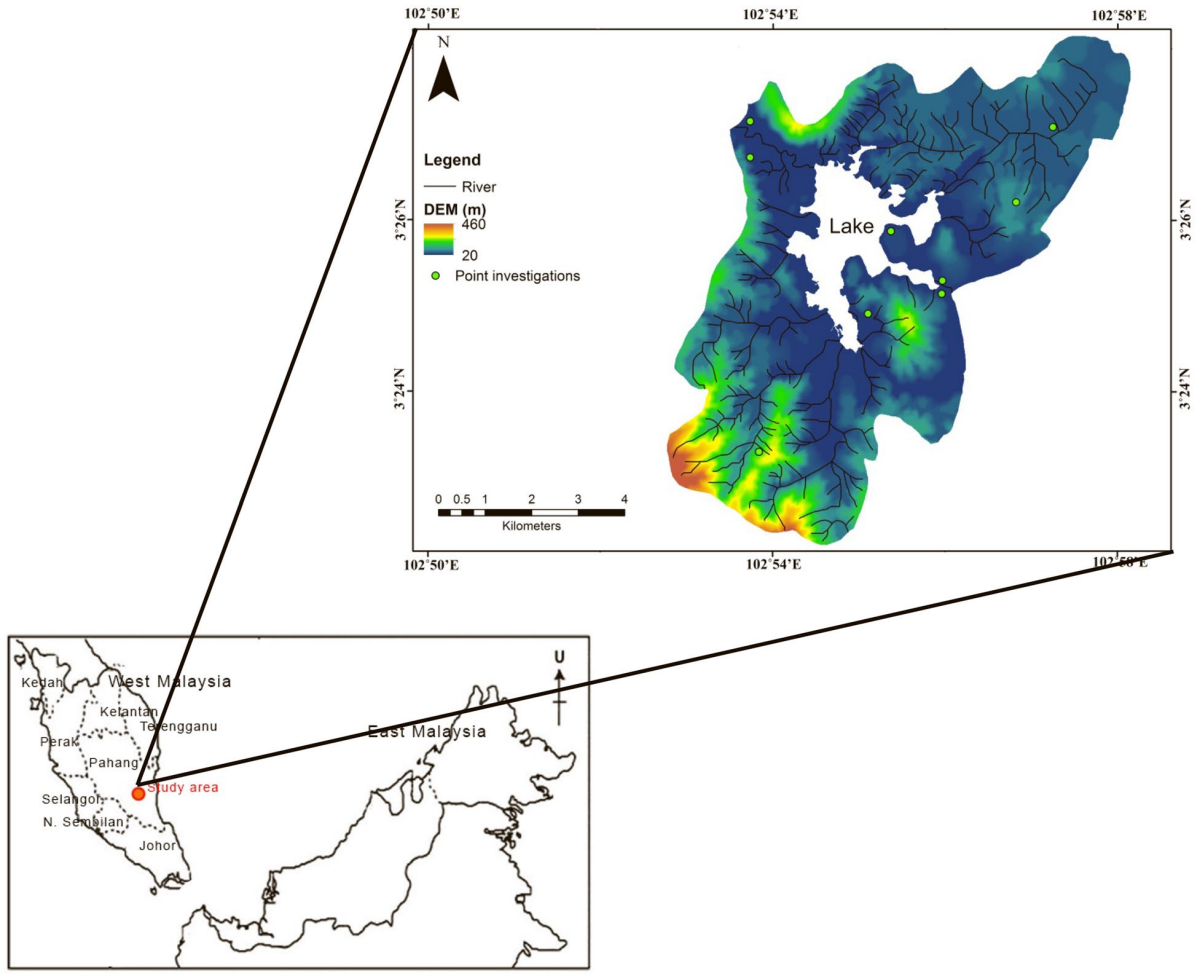
Study area with points of soil sampling presented on the digital elevation model (DEM) map

This research was carried out in the Chini lake basin region encompassing 52.89 km^2^ in Pahang State situated on the Eastern coast of Peninsular Malaysia. The study region is located between the latitude 3° 23′ N–3° 28′ N and longitude 102° 53′ E–102° 58′ E (Fig. 1). The clime of this region was categorized into two types of monsoon; southwest and northeast monsoons (Hin and Othman 2020). The average annual precipitation was about 2500 mm and the temperature was about 28 °C. The study region was distinguished by various land-use classes including forest, agricultural, urbanized, and bare and mining areas.

### Data acquisition and analysis

To estimate the K factor values, the soil properties data were obtained from soil sampling in the field and laboratory analysis of soil samples. The random soil sampling method was conducted in ten points around the study area which covered distinct soil series (Fig. 1). Landsat 8 images and digital elevation model (DEM) were obtained from the United States Geological Survey (USGS) data portal. The satellite images consisted of three dates for current scenarios of soil erosion rate; Landsat 8 OLI Level-2 Product on 7th September 2019, 22nd August 2020, and 12th September 2021. These images have undergone atmospheric correction analysis using ArcGIS 10.2 software.

The WorldClim website was widely used to obtain downscaled CMIP6 annual precipitation parameters. This study used the historical precipitation of the latest published CMIP6 GCMs. In the CMIP6, the representative concentration pathways (RCP2.6, RCP4.5, RCP6.0, and RCP8.5) from the previous CMIP5 model have been renewed to the shared socio-economic pathways (SSP2.6, SSP4.5, SSP6.0, and SSP8.5). The CMIP6 model with the updated improvements such as improved physical processes, land use planning, and model parameterization was marked to give the best simulation of future climate. In this study, a HadGEM3–GC31–LL model of GCMs each for precipitation was chosen from the CMIP6 database for the period 2020–2080. The period 2020–2080 was considered, since the CMIP6 GCMs have more than 20-year baseline period. The GCMs were selected according to the availability of the SSP (at least one SSP), GCMs among precipitation parameters, and the availability of precipitation data for the study area. Furthermore, the CMIP6 GCMs have not been studied for Malaysia until this time, thus we chose to construct this climate model for historical soil erosion events.

Compared to the CMIP5, the CMIP6 model has finer model resolution and updated the processes of physical (Stouffer et al. 2017). we applied one of the CMIP6 models (HadGEM3–GC31–LL), because it showed a better performance than the other models for seasonal average temperatures in terms of bias. Cui et al. (2021) analyzed the spatial distributions of the observed and multimodel ensemble for mean annual temperature and mean summer temperature. According to their study, the CMIP6 models exhibited a similar spatial pattern.

A previous study has revealed that the CMIP6 model showed significantly higher climate sensitivity than the prior CMIP5 model (Zelinka et al. 2020). In this study, the HadGEM3–GC31–LL model was also employed for computing R factor values. For an observation of the effects of future climate change on soil erosion by water, the results of the current climate and predicted climate change based on the HadGEM3–GC31–LL model and some SSP (SSP2.6, 4.5, and 8.5 in 2040–2060 and 2060–2080) were applied in our estimation of the rainfall erosivity factor.

### Soil erosion calculation

In the tropical regions of Malaysia, soil erosion by water was one of the great composite ecological issues impendent agricultural sector and human activities. The soil erosion analyses in the Chini Lake Basin have been investigated by previous works concerning the erosion of water (Gupta and Kumar 2017; Teng et al. 2018). However, there were no studies that have applied the analysis of soil erosion under recent CMIP6 scenarios in the Chini Lake Basin, even, in the Southeast Asian region. Hence, this is a novel study to estimate current and future soil erosion potential under CMIP6 scenarios in this region. Our study did not assess wind erosion, but we suggest for next future studies shall integrate both wind and water erosions. Some criteria of RUSLE (R, K, LS, C, and P) were obtained from in situ measurement and earth observation data. Afterward, a raster map was simulated in geographical information system software to evaluate the spatial–temporal of soil erosion rate in the Chini lake basin area. With the incorporation of improved techniques in estimating soil erosion with current data sources, the produced soil erosion map could represent a higher accuracy, compared with other previous works (Menshov et al. 2018; Krasa et al. 2019).

The RUSLE method was applied for calculating and estimating soil erosion rate in this study. It was one of the best popular techniques for soil erosion analysis and mapping (Kebede et al. 2021). The soil erosion analysis using RUSLE was according to the following equation:

1 $$ {\text{Soil erosion rate }} = \, \left( {{\text{R }} \times {\text{ LS }} \times {\text{ K }} \times {\text{ P }} \times {\text{ C}}} \right) $$

Soil erosion rate was stated in t/ha year; R was rainfall erosivity; LS was a topographical factor; K was soil erodibility, P was support practice; C was land cover management factors.

The rainfall erosivity factor represented the total energies of water drops that markedly influenced the soil aggregate stability and promoted soil erosion (Lee et al. 2021). This factor was determined from annual rainfall data using a combination formula from Morgan (2009) and Roose (1977) as specified in Eqs. (2–4):

2 $${R}_{\mathrm{morgan}}= \frac{(9.28\rho - 8838.15) \times (75)}{100}$$

3 $$ R_{{\text{roose}}} = \, 0.{5 } \times \, \rho \, \times { 17}.{3} $$

4 $$R = \frac{({\mathrm{R}}_{\mathrm{morgan}} + {\mathrm{R}}_{\mathrm{roose}})}{2}$$

*R* was the rainfall erosivity factor (MJ mm/ha hr year), and ρ indicated the rainfall yearly (mm). The rainfall erosivity factor was analyzed for two distinct scenarios to calculate the future and current R factors. Rainfall erosivity values were acquired by calculating the annual *R* values from 2019 to 2021, and they were used as delegations for the current period. In the current period, we would like to investigate the effect of the COVID-19 pandemic where in 2020 there was a restriction period implemented in the country so it would be interesting to find out how soil erosion when human activities were suspended. Based on the COVID-19 studies, the virus would keep on growing around the world until the 2020 year when no mitigation actions were conducted (Rendana and Idris 2021). While, for future climate estimation, there were two distinct mean values were chosen for the rainfall erosivity factor. The first was from 2040 to 2060 and the other was from 2060 to 2080 periods.

The erodibility of soil indicated soil structure vulnerability to being dispersed by water drops and carried by runoff (Jiang et al. 2020). The soil erodibility values were determined using soil sampling and laboratory analysis according to the standard method, the formula was shown in Eq. (5) by Tew (1999):

5 $${K}_{\mathrm{factor}}=\frac{(2.1 \times {10}^{-4} (12 -\mathrm{ OM}) {(N1\times \mathrm{ N}2) }^{1.14} + 3.25 (\mathrm{S }- 2) + 2.5 (\mathrm{P}-3)) }{(100 \times 7.59)}$$

*K*_factor_ was the erodibility of soil (t ha hr/ha MJ mm), N1: clay (%) + very fine sand (%), S: soil structure, N2: clay (%) + very fine sand (%) + sand (%), and P: hydraulic conductivity (cm/hr). Thereafter, each polygon layer containing soil series was specified a K value in the GIS setting. In this study, we also compared our K values with K values from the Department of Irrigation and Drainage of Malaysia (2010) and used them for covering nonsampling areas.

The steepness and length of the slope had crucial components in soil loss. It indicated potencies of topographical factors in soil loss and runoff events (Brychta and Brychtová 2020). Topographical factors were determined by a formula from Wischmeier (1975) in the following equation:

6 $$ {\text{LS}}_{{\text{factor}}} = \left( {\frac{L}{22.13}} \right) \times {\text{m}} \times (0.065 + 0.046s + 0.0065s^2 ) $$

L was flow accumulation (the upper slope region to a specified pixel) with a size of a pixel of 30 m. Flow accumulation was generated from DEM; 0.6 was chosen as the m value, because slope > 12%; s represented percent slope.

Management of land cover or C factor had a prominent role in soil erosion analysis by covering the upper layer of soil from the impact of water drops. Several studies have found soil erosion was highly associated with vegetation cover (Nikolic et al. 2019). The classification of the distinct land cover classes was carried out using three Landsat 8 satellite images acquired in 2019, 2020, and 2021. The Landsat images were obtained from the United States Geological (USGS) Earth Explorer. The selection of the Landsat satellite images dates depended on the image quality based on the percentage of cloud cover. Each image was georeferenced to the WGS 84 datum and Kertau RSO Malaya Meter coordinate system.

**Fig. 2:**
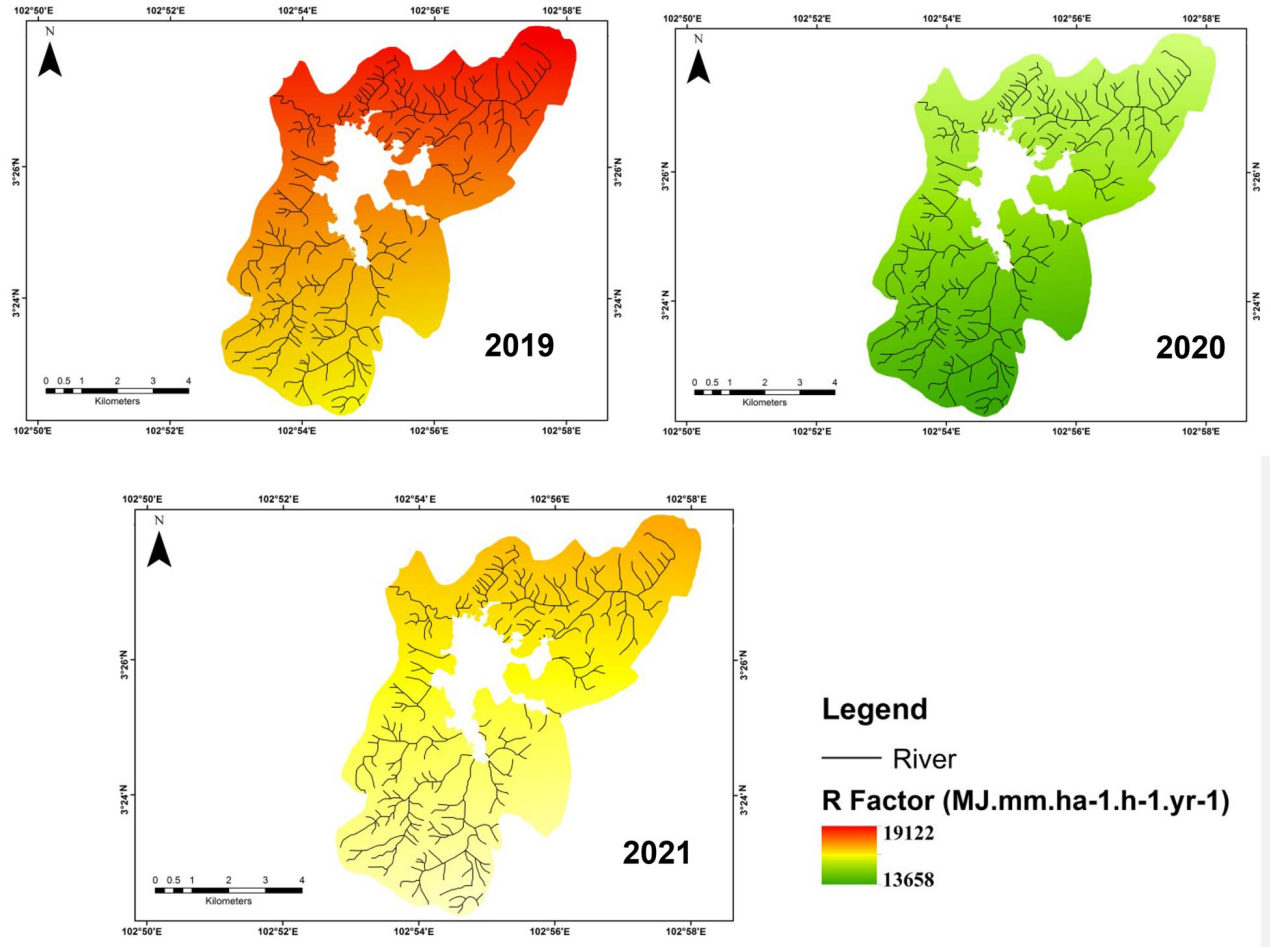
R factor distribution (2019; 2020; 2021) in Chini lake basin area

A complex pre-processing such as geo-referencing, layer stacking, mosaic, and atmospheric correction is conducted to ortho-rectify the satellite images and remove the effect of the atmosphere on the reflectance values of images taken by satellite. In this study, the supervised classification was performed to classify the land cover/use. The supervised classification based on (Shakya et al. 2018) is where the user develops the spectral signatures of known classes (i.e., urban and forest) and then the ArcGIS software will set values in each pixel in the image to the class that its signature is most proportionate. The supervised classification was applied after the user creates the area of interest (AOI) or training classes. The training sites were chosen in accordance with the field data or sampling points (Fig. 1). Finally, the images were categorized into six classes as shown in Table 1. The output of the Kappa coefficient for accuracy analysis showed a good value of 0.85 and total accuracy of around 88%. Formulas of the Kappa coefficient and total accuracy are shown in Eqs. 7–8:

7 $$ \frac{{N\sum_{i = 1}^r {x_{ii} } - \sum_{i = 1}^r {\left( {x_{i + } } \right)\left( {x_{i + } } \right)} }}{{N^2 - \sum_{i = 1}^r {\left( {x_{i + } } \right)\left( {x_{i + } } \right)} }} $$

8 $$ {\text{OA}} = \left( \frac{1}{N} \right)\sum_{i = 1}^r {n_{ii} } $$

where *r* was the number of rows in the matrix, *x*_*i*+_ and *x*_*ii*_ were the marginal totals of row *r* and column *i*, *X* was the number of observations in row *i* and column *i*, *N* was the total number of observations, and *n*_*ii*_ was the number of correctly classifed pixels (Fig. 2).

**Table 1:** P and C factors with recent total area land-use in Chini lake basin area. *Source*: Department of Irrigation and Drainage of Malaysia (2010), Morgan (2009)

Land use	C factor	P factor	Area (km^2^)	Area (%)
Bare land	1.00	0.10	3.2	6
Agricultural area	0.20	0.40	8.9	16.7
Urbanized area	0.25	0.70	5.4	10.2
Forest/tree	0.03	0.10	35.5	67.1

The conservation practice or *P* factor indicated soil erosion from upper and below slopes under certain conservation uses. For example, terracing, contour, strip cropping and influence the runoff course and change flow distribution (Wen et al. 2021). *P* factor values were specified based on the recent land-use map and recommended by the Department of Irrigation and Drainage of Malaysia (2010) and Morgan (2009) (Table 1).

Spatial variation of soil erosion was obtained by calculating all the RUSLE factors to produce current and future soil erosion maps. In the matter of future soil erosion, K, P, and LS factors were calculated in the same recent scenario, *C* values were computed in a mean of C_2019_, C_2020_, and C_2021_, whilst the *R* factor was calculated as a mean for two distinct periods; *R*_2040-2060_ and *R*_2060-2080_. Therefore, the future soil erosion map could be stated in the following equation:

9 $$ {\text{SSP2}}.{6},{\text{ SSP4}}.{5},{\text{ SSP8}}.{5}:{\text{ A }} = {\text{ R}}_{{\text{mean}}} \times {\text{ K }} \times {\text{ LS }} \times {\text{ C}}_{({\text{mean}})} \times {\text{ P}} $$

### Statistical analysis

This study calculated average values for RUSLE factors and total soil erosion values by extracting values in each pixel raster map using ArcGIS software. Eventually, the relationship between total soil erosion and each RUSLE factor was analyzed using Pearson correlation analysis in IBM SPSS Statistic 21 software.

## Results and discussion

### Factors impacting soil erosion in the Chini Lake Basin

Based on our results, the erodibility of soil values greatly differed. It ranged from 0.001 to 0.039 ton ha hr/ha MJ mm. This divergence could be affected by soil type and land use in the study area. For instance, the K factor values in dense areas such as forests and secondary forests showed low K factor values (southern, southeastern, and southwestern parts) while the agricultural and urbanized areas showed high K factor values (northern, northeastern, and northwestern parts). This was consistent with a previous study by Hateffard et al. (2021) that found the erodibility factor was mainly high in the agricultural area from 0.30 to 0.44 ton ha hr/ha MJ mm. The K factor values tended to reduce in regions with high elevation. The K factor values in this study were obtained from the calculation of soil properties based on data collected from field sampling activity. The K values from the laboratory work were matched with the K values from the Department of Irrigation and Drainage of Malaysia (2010). There was about a 95% match of the K values based on our calculation and the Department of Irrigation and Drainage of Malaysia (2010), thus it showed that the K values could be used in this study. According to our results, the mean K factor was 0.01 t ha hr/ha MJ mm, with the mean organic matter was 3.4%, and clay percent was 80.4%, which led to increasing the K values in this study. However, Our K values were consistent with the other studies. For example, in Iran, the mean K factor was 0.01 t ha hr/ha MJ mm (Ostovari et al. 2019), in India, the K values were 0.01–0.1 t ha hr/ha MJ mm was found (Jena et al. 2018), and the mean K factor was 0.01 t ha hr/ha MJ mm in China (Teng et al. 2019).

**Fig. 3:**
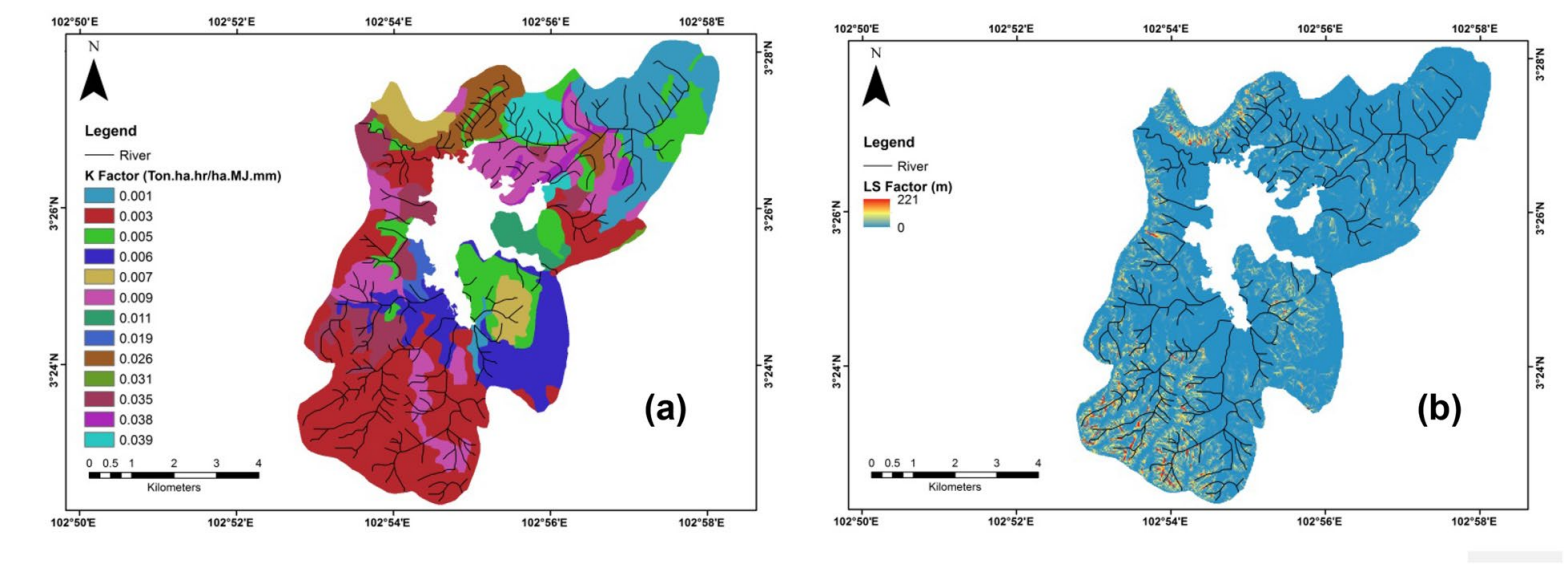
Spatial variation of (**a**) K factor and (**b**) LS factor in Chini lake basin area

The average value of the LS factor (slope length) was 4.51 m (Fig. 3). As a whole, the LS value in this study ranged from 0 to 221 m. The LS value was greater in the southern, southwestern, and northwestern parts of the study area than in the northern, northeastern, and southeastern parts which were distinguished by low slope and runoff rates. Although the dominant P factor was classified as forest areas with more than 60% of the study area. Meanwhile, the bare lands would contribute to a high P value due to high erosion potential, although these areas had less than 10% (Fig. 5). The topographical factor was estimated according to the clustering of the slope raster map. This factor was considered to be the most affecting factor for erosion headway. This result was in line with Zhang and Wang (2017) who also found that this factor was very associated with the increase in soil erosion values. Another study by Pham et al. (2018) stated the P and C factors could minimize the soil erosion values, by planting vegetation cover and building stone walls. In a study that was carried out at complex hillslopes, Sabzevari and Talebi (2019) revealed the greatest erosions were observed in slopes (distended discrete type), while the lowest erosions were associated with slopes (sunken discrete type). As a whole, analyzing the map of LS factor with the soil erosion map showed that raising the length of the slope, frequency, and level of soil erosion also was raised was the same as the finding of Qin et al. (2018).

**Fig. 4:**
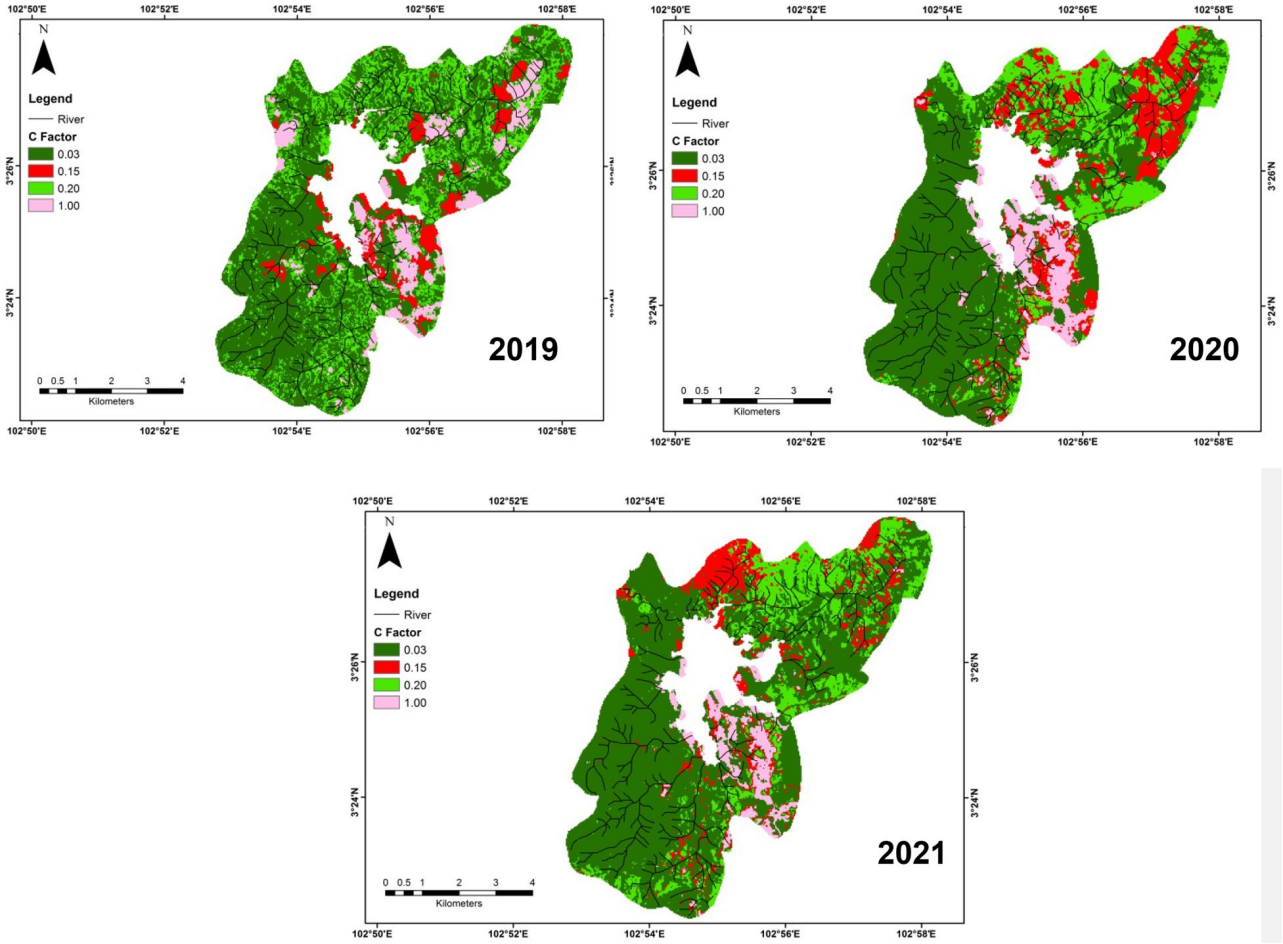
Cover management (C) factor pattern (2019; 2020; 2021) in Chini lake basin area

Figure 4 exhibits C factor maps in several years; 2019, 2020, and 2021. There was no notable distinction in C value in study periods. However, it was mainly different during 2019. Hence, the land cover during 2019 was the most susceptible to a raising erosion rate. There was a bit of difference between the C factor map in 2019 and 2020 where the clouds have become obstacles to analyzing what land covers below them. This was the reason we obtained more urban and bare areas during those periods, because the reflectance value of clouds gave identical signatures with urban or bare areas. The intensive land-use conversion could highly influence the C factor values (Almagro et al. 2019). During the current scenario (2019–2021), C factor values in 2019 showed a significantly different from 2020 and 2021, which could be caused by the increase in open land areas for mining and agricultural activities. To support this notion, we compared the land use obtained in this study with the land use data from the Department of Agriculture. It showed that the Chini lake basin experienced a great change during that period. All surrounding the Chini lake, specifically in the northwestern area, the forest covering the land. In the northeast of the basin, the areas were first covered by forest, but recently the forest was mostly cut down, only left orchards and shrubs. Besides, the sides of the basin were majorly converted to oil palm areas. The oil palm areas expanded until the southeastern side of the basin. They occupied about 70% of the basin area. In the center of the basin, there were abandoned and activated mining sites. The use of land for this activity increased year after year based on the ore resource demand.

In addition, the prior studies also reported that the study area had great potential for iron and barite ores thus it was conducted mining activities, especially in the southeastern parts of the study area. While, in the northern parts, the oil palm plantation has greatly developed in the study area due to the suitable soil for oil palm growth. The C factor was not different from the R factor as it was also prone to the regions under tropical conditions and anthropogenic effects (Jiang et al. 2017). Therefore, both factors contributed to the complexity and increment of soil erosion rate in the study area. Results of our study also found that non-agricultural regions such as urban areas and bare areas also experienced dynamic changes thus they could be considered for increasing soil erosion. Swarnkar et al. (2018) reported that there was an agreement in the soil erosion science assemblage regarding the exact C and P factor values due to the impacts of different slope-preservation techniques. Hence, we suggested the authority concerned to the areas where these two factors exhibited significant changes from the current period to the predicted period to evade a permanent loss of soil fertility or flood events, and tremendous sediments flowing to rivers.

**Fig. 5:**
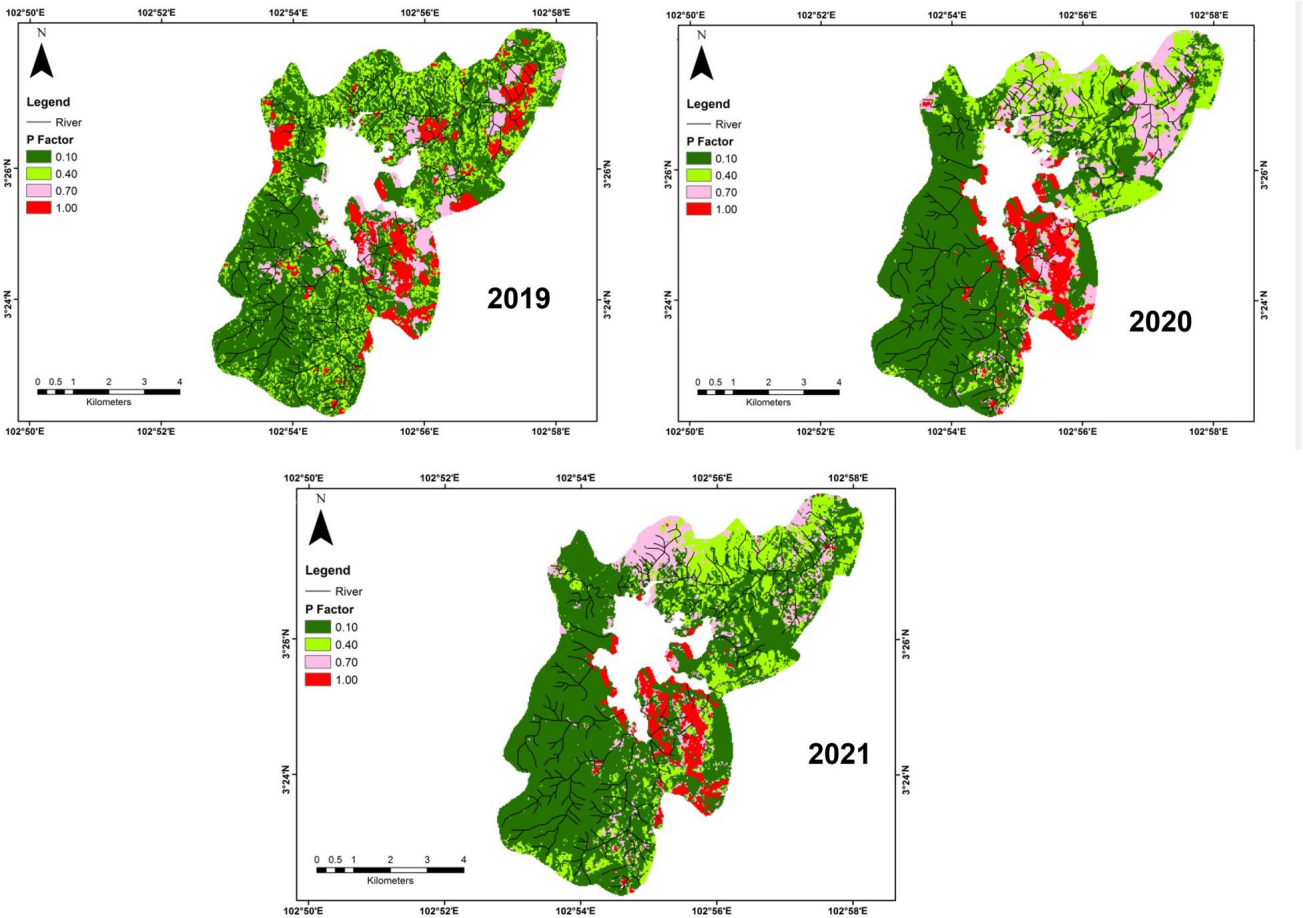
P factor pattern (2019; 2020; 2021) in Chini lake basin area

In this study, we used three R factors (rainfall erosivity) values from 3 years of study periods (2019, 2020, and 2021) (Fig. 2). The highest R factor values were observed in 2019 followed by 2021 and 2020. The annual mean R factors in 2019, 2020, and 2021 were 18,777, 13,861, and 16,504 MJ mm/ha hr year. The highest R factor was majorly observed in the northern to northeastern parts of the study area that related to lower terrain (Fig. 5).

### Current soil erosion potential scenario

**Fig. 6:**
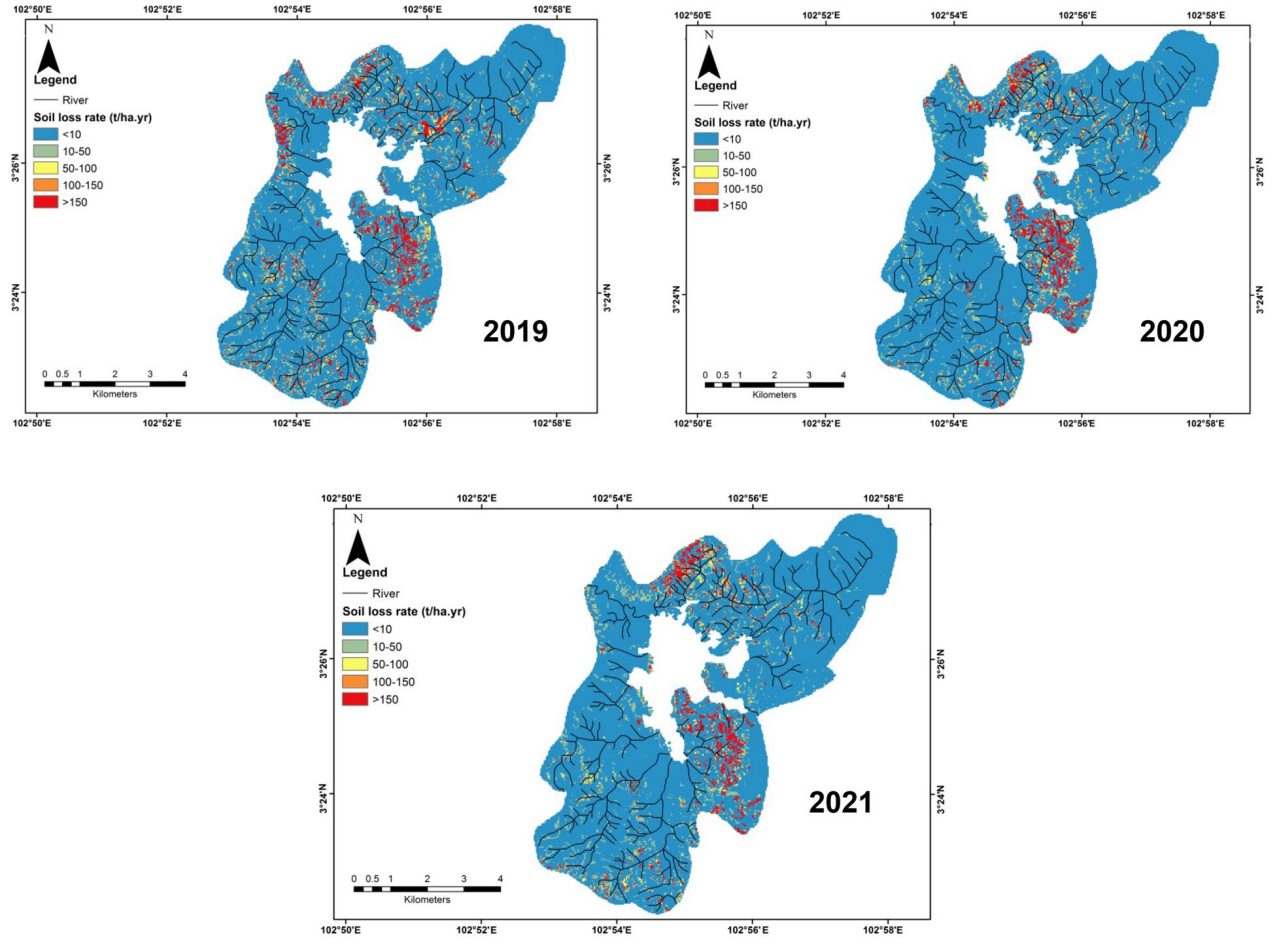
Spatial distribution of soil erosion rate (2019; 2020; 2021) in Chini lake basin area

Figure 6 shows the mean predicted soil erosion rate in the Chini lake basin area in 2019, 2020, and 2021 was about 73.96, 40.18, and 37.12 t/ha year, respectively. According to this result, the COVID-19 period has significantly decreased soil erosion rates, especially during the restriction period (2020) and post-restriction period (2021). The closure of human activities has dropped the number of sediments that originated from the tourism and mining sites around the study area which were carried by runoff events and finally polluted the water body. This was consistent with other studies that also found there was an improvement in water quality during the lockdown period in India (Yunus et al. 2020), China (Liu et al. 2022), and Turkey (Tokatlı and Varol 2021). The southeastern part of the study area was more vulnerable to soil erosion when this area recorded more than 150 t/ha year of erosion, otherwise to the northern areas less than 10 t/ha year. Table 2 depicts the percentage of areas that influenced soil erosion classes during the current scenario. The current erosion rate in major areas of the basin area was at class 1 (< 10 ton/ha year) (Table 2). Table 3 exhibits the correlation analysis between RUSLE values and soil erosion values in the study area. This was found LS factor, C, and P factors correlated with soil erosion rate. In contrast, R and K factors had less correlation with soil erosion rate in the study area.

**Table 2:** Percentage of soil erosion classes under 3-year periods in the study area

Soil erosion class (t/ha year)	Years		
	2019 (%)	2020 (%)	2021 (%)
Class 1 (<10)	84.07	86.68	87.57
Class 2 (10–50)	7.78	6.45	6.49
Class 3 (50–100)	2.06	1.69	1.45
Class 4 (100–150)	1.07	0.99	0.86
Class 5 (>150)	5.03	4.19	3.63

Class 1: very low; class 2: low, class 3: moderate, class 4: high, and class 5: very high

**Table 3:** Pearson’s correlation between soil erosion rate and RUSLE factors

	RUSLE factors				
	Rainfall erosivity	Topography	Soil erodibility	Support practice	Land cover
Correlation value (*r)*	0.94**	0.70*	− 0.48	0.74*	0.75*
*p* value	0.01	0.02	0.06	0.01	0.01

^*^Correlation is significant at the 0.05 level (two-tailed)

Spatial analysis of soil erosion could assist to calculate the annual soil loss values that were greater than 10 t/ha year. The results showed that the southeastern and northwestern areas were the most influenced from the 2019 to 2021 periods. These regions were distinguished by bare areas and steep slopes areas. Our study observably ensured that soil loss was able simply influenced by diverse land cover types. The C factor map exhibited that bare areas were mostly found in the southeastern areas that had the greatest values of soil erosion, where the LS factor varies from 0 to 121 (Fig. 3b). In contrast, agricultural areas governed in the low slope regions with LS factor varied from 0 to 13, which reduced the soil erosion process. Based on this notion, Table 3 obtains the greatest relationship between the LS factor and soil erosion (*r* = 0.70, *p* < 0.05), which indicated the crucial role of the length of the slope in promoting soil erosion. Considering the erosion process in the bare areas and as followed by some urban areas revealing the greatest soil erosion, prosecuted greater control and preservation. Hence, those areas should be taken into in the next land preservation strategy in light of topographical factors as a prominent key to climate shift and soil erosion frequency (Borrelli et al. 2017). This output totally assured the finding of a study by Saco et al. (2018) who explained that higher slope regions with sparse vegetation could contribute to greater erosion occurrences. Bare areas have exhibited the highest soil erosion values in the current scenarios situated on steep slope areas.

To minimize the soil erosion rate in basins, humans could apply soil and water conservation measures within the basins. Sun et al. (2019) investigated the application of large-scale ecological restoration and it led to a notable reduction of sediment load from the Chinese Loess Plateau into the Yellow River. It was the same with another study in the Zhou River Basin where the sediment load and streamflow of the basin were reduced by 80% and 50%, respectively. The landscape engineering actions were primarily responsible for both factors decreased (Sun et al. 2020a). In addition, a higher risk of high sediment load frequently occurred during heavy rainfall and within small basins (Sun et al. 2020b).

### Predicted future soil erosion potential scenario

**Fig. 7:**
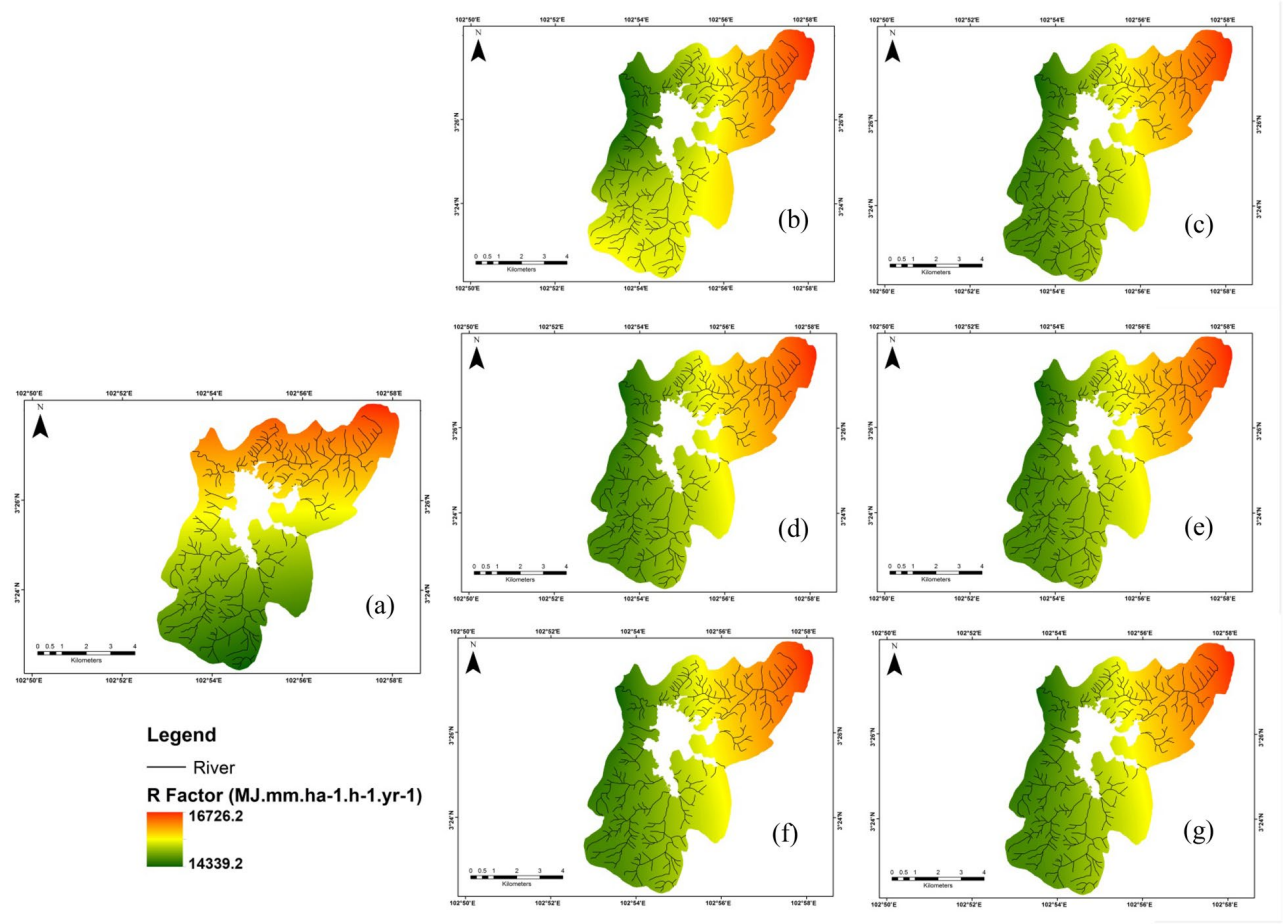
Estimated changes in R factor according to the HadGEM3–GC31–LL model and SSPs for distinct scenarios (**a**) baseline 2019–2021, (**b**) SSP2.6 (2040–2060), (**c**) SSP2.6 (2060–2080), (**d**) SSP4.5 (2040–2060), (**e**) SSP4.5 (2060–2080), (**f**) SSP8.5 (2040–2060), (**g**) SSP8.5 (2060–2080)

**Fig. 8:**
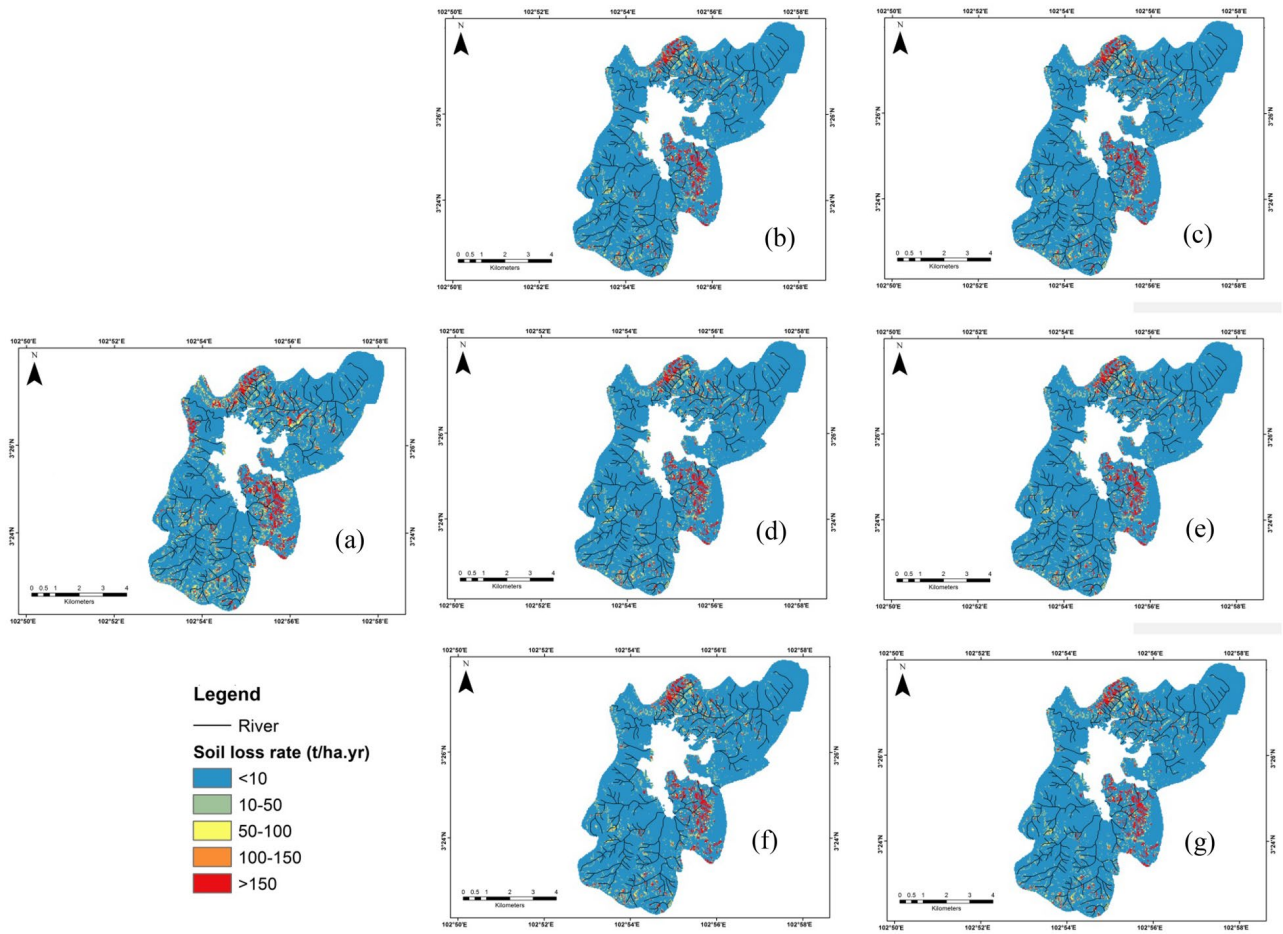
Estimated future soil erosion changes under distinct scenarios of SSPs (**a**) baseline 2019–2021, (**b**) SSP2.6 (2040–2060), (**c**) SSP2.6 (2060–2080), (**d**) SSP4.5 (2040–2060), (**e**) SSP4.5 (2060–2080), (**f**) SSP8.5 (2040–2060), (**g**) SSP8.5 (2060–2080)

Several scenarios of estimated R value were analyzed from a new generation of climate design, or CMIP6 for periods of 2040–2060 and 2060–2080. Comparing the baseline and predicted R values computed from annual rainfall data (2019–2021) with the predicted R values obtained from three “Shared Socioeconomic Pathways” (SSPs) is found in Fig. 7. The greatest R factor values were mostly found from the northwestern to the northern part of the basin region. The same distribution was shown in the predicted R (Fig. 7). Figure 8 reveals the alterations between current and predicted soil erosion under climate change scenarios of SSPs. This was estimated the areas with greater R factor values were situated from the northern to the northeastern part of the study area.

The current and downscaled R factor were calculated and mapped according to national rainfall stations (for current rainfall data), and rainfall data acquired from WorldClim data (for predicted rainfall data). The precise modeling of current rainfall erosivity values produced the best outputs in predicting soil erosion, specifically in regions with poor annual rainfall values and also greatly driven by climate factors. These outputs could deliver new notions to evaluate the presence of gullies and rills erosion processes, because they were very prone to the alteration of precipitation scheme and human activities effects (Kou et al. 2020).

The predicted soil erosion values showed a very high soil erosion (> 150 t/ha year) mostly found in the northwestern and southeastern areas of the basin region under SSP scenarios (Fig. 8). This result exhibited the same spatial variability of soil erosion as the current scenario. This future model revealed these similar areas would be exposed to speed the rate of soil erosion if appropriate land conservation policies were not applied. Specifically, areas of very high erosion values (> 150 t/ha year) reduced to 3.5% in the SSP2.6 (2061–2080) and 5.5% in the SSP8.5 (2061–2080) (Table 4). Spatial diversity between future RUSLE under distinct SSPs scenarios and current RUSLE (2019–2021) exhibited a slightly decrease erosion rate in most areas of the basin region.

**Table 4:** Zonal statistical (average t/ha year) for each soil erosion classes under baseline and estimated SSP scenarios

Scenario	Time series	Mean soil erosion rate (t/ha year)	Percentage soil erosion class (%)				
			Class 1 (<10 t/ha year)	Class 2 (10–50 t/ha year)	Class 3 (50–100 t/ha year)	Class 4 (100–150 t/ha year)	Class 5 (>150 t/ha year)
Baseline	2019–2021	50.42	86.1	6.9	1.7	1.0	4.3
SSP2.6	2040–2060	33.30	88.3	6.0	1.5	0.8	3.4
	2060–2080	34.52	88.1	6.1	1.5	0.8	3.5
SSP4.5	2040–2060	35.90	87.8	6.3	1.5	0.8	3.6
	2060–2080	36.15	87.8	6.3	1.5	0.8	3.6
SSP8.5	2040–2060	35.36	87.9	6.2	1.5	0.8	3.5
	2060–2080	33.46	88.3	6.0	1.5	0.8	3.4

To assess the effect of future climate shifts on soil loss sensitivity, data obtained from CMIP6 by some predictions of CMIP6–SSP were applied in computing R values in the future. The used method in our work was in line with a previous study by Hateffard et al. (2021) who found the predicted rainfall values would definitely cause soil erosion rate changes. The predicted soil erosion values were estimated in the study region based on the regional climate change that concerned 3 scenarios of SSPs. These results showed that the southeastern and northwestern parts were the most susceptive to climate change factors, particularly in SSP8.5 that in line with the pathway in enormous quantities of greenhouse gas emissions. The high soil erosion values were situated majorly in bare areas with steep slopes, thus, it would be highly influenced by alterations of rainfall distributions for climate traits in tropical regions. The raising in rainfall intensities over the study region would induce a higher risk of soil erosion through runoff events from steep slope areas. Therefore, the estimation of future soil erosion was very prominent to give authority with suitable tools for arranging immediate actions against distinct probable soil erosion scenarios. Panagos and Katsoyiannis (2019) highlighted the significance of mapping soil erosion on a distinct scale for protection action and policy arrangement. While Maurya et al. (2021) underlined the significance of the application of preservation plans according to SSP2.6 and SSP8.5 scenarios.

In light of all the factors, it was clear that soil erosion in the southeastern and northwestern regions that were predominated by bare areas, greater rainfall, and the steep slope was experiencing serious erosion and had the greatest risk of soil erosion. Based on the geological aspects in the study region, we assumed that the geological had a prominent part in soil erosion occurrences, however, was majorly represented in the form of the erodibility of soil. The fine-grain clastic sediments which encompassed the northern to southern regions of the study areas were related to the minimal susceptibleness of K values. In the lower hill area, sedimentation was the primary feature thus soil erosion has sustained a little quantity as well. There were two areas of the study region obtained great prone, the first was in the mining regions that indicated enormous erosion was clear, and the second was situated in agricultural regions with steep slopes with proof of dispersion and gully erosion kinds. The result of this study can be applied to arrange control actions of valuable freshwater lake basin areas in the tropical regions and implement this area became a high priority for ecological and biological conservation purposes. In addition, soil erosion could be minimized by preserving vegetation cover, and diminishing soil disruption by agricultural activities (Eswaran et al. 2019).

The future prediction of soil erosion (36.15 t/ha year) were found high for SSP4.5 during 2060–2080. Whereas, the lower values (33.30 t/ha year) was found for SSP2.6 during 2040–2060 (Table 4). There was a reduction of soil erosion classes areas (classes 2–5) observed at 0.9%, 0.2%, 0.2%, and 0.9%, respectively, compared to the baseline year (2019–2021). However, the increment was observed as 2.2% for class 1 compared to the baseline year (2019–2021) which indicated that there was an improvement in soil quality in the study area (Table 4). Overall, we predicted the future soil erosion values in this area would decrease by around 33.9% compared to the baseline year (2019–2021). In addition, we assumed the future climate change might affect the changes in the underlying surface. Based on our results, future climate might play the dominant role in rainfall change, compared with that of other RUSLE factors. The significant changes in rainfall were the main navigating force of soil erosion change in the Chini lake basin. Thus, this study suggests that rainfall is a prominent factor that should be more considered in the resulting soil erosion change, since the studied basin was situated in the tropical region with a high amount of rainfall.

**Fig. 9:**
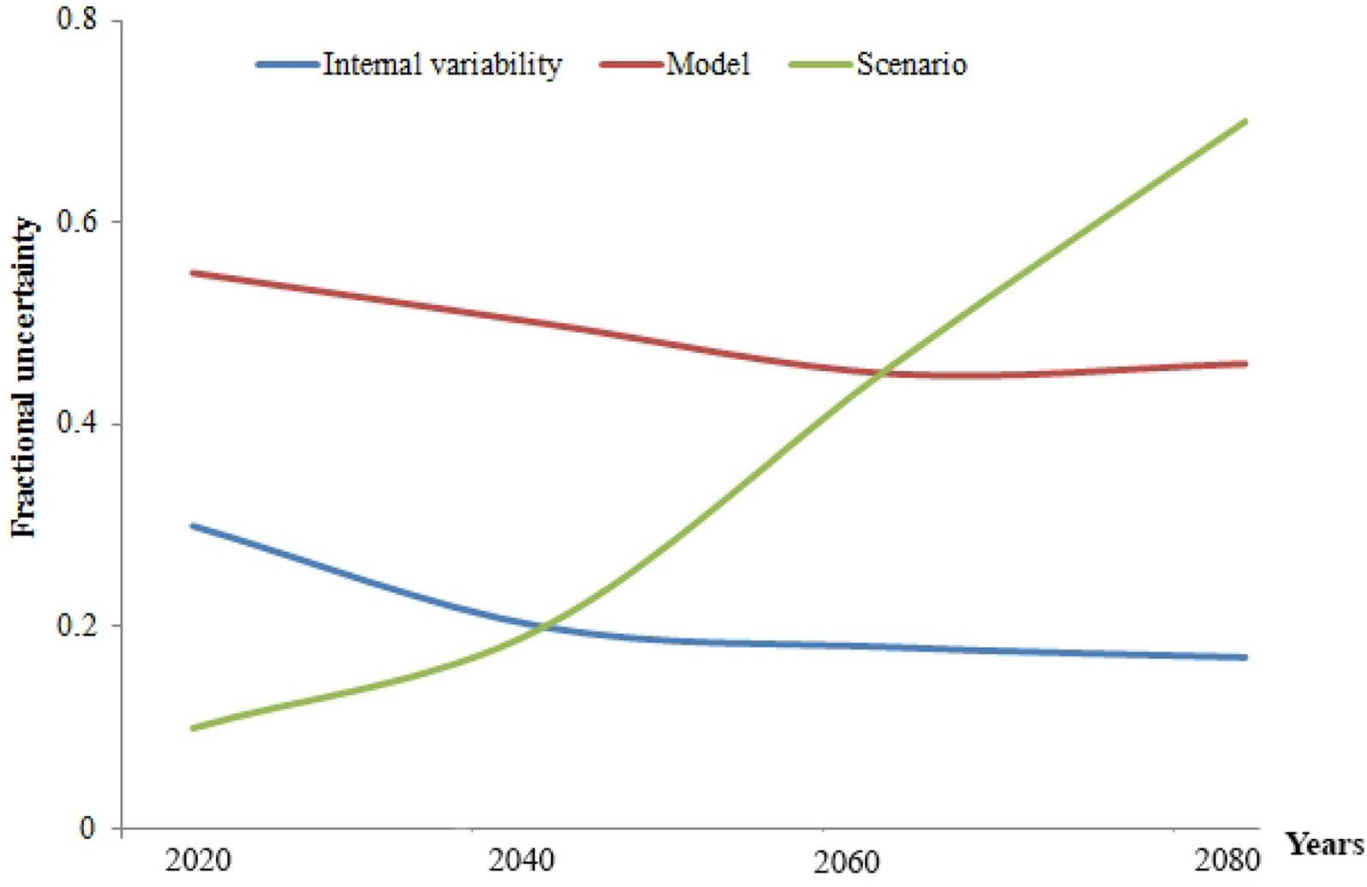
Fractional uncertainty with the 90% confidence level divided by the average estimation over the study area. Different uncertainties were computed for internal variability, model and scenario parameters

Uncertainty in the model prediction was analyzed using three main parameters, such as internal variability, model uncertainty, and scenario uncertainty. Fractional uncertainties in the decadal annual soil erosion rate over the Chini catchment area are depicted in Fig. 9. The uncertainty of the annual soil erosion rate dominantly came from model and scenario uncertainties, and the total fractional uncertainty showed a minimum of about 2040 related to the contributions from scenario and model uncertainties.

## Conclusions

Soil erosion is one of the climate hazards that can deteriorate the economy and livelihood of a country. Based on the global climate index, Malaysia is ranked among the two hundred nations most threatened by climate change during 2019–2021. The integration of remote sensing data and climate model projection is very useful for studying geological phenomena and active processes, such as soil erosion. Our study concluded that there was a reduction of future soil erosion values of around 33.9% compared with the baseline year (2019–2021). The rainfall erosivity factor greatly affected the changes in soil erosion in the study area, since this area was categorized as a tropical region. The highest future soil erosion values were found in SSP4.5 during 2060–2080. However, the lowest values were found in SSP2.6 during 2040–2060. The finding of this study could contribute to achieving the United Nations' 2030 Agenda for Sustainable Development.

## Data Availability

The data that support the findings of this study are available from the corresponding author, MR upon reasonable request.
